# Anti-inflammatory effect of different curcumin preparations on adjuvant-induced arthritis in rats

**DOI:** 10.1186/s12906-021-03207-3

**Published:** 2021-01-21

**Authors:** Ieva Rinkunaite, Egidijus Simoliunas, Milda Alksne, Dominyka Dapkute, Virginija Bukelskiene

**Affiliations:** 1 Institute of Bisochemistry, Life Sciences Center, Vilnius University, Sauletekio av. 7, LT-10257 Vilnius, Lithuania; 2 Institute of Biosciences, Life Sciences Center, Vilnius University, Sauletekio av. 7, LT-10257 Vilnius, Lithuania

**Keywords:** Curcumin, Microencapsulation, Adjuvant-induced arthritis, Inflammatory cytokines

## Abstract

**Background:**

Curcumin, a natural polyphenolic substance, has been known for more than two millennia as having strong anti-inflammatory activity towards multiple ailments, including arthritis. The main drawback of curcumin is its poor solubility in water, which leads to low intestinal absorption and minimal bioavailability. In this study, we aimed to compare the anti-arthritic in vivo effect of different curcumin preparations – basic curcumin extract, micellar curcumin, curcumin mixture with piperine, and microencapsulated curcumin.

**Methods:**

Arthritis was induced in Wistar rats by complete Freund’s adjuvant, and the severity of arthritis was evaluated daily using the arthritis score system. Curcumin preparations were given to animals per os daily for 20 consecutive days, starting at 6th day after arthritis induction. To determine the inflammatory background, pro-inflammatory cytokines were determined using the ELISA test. In addition, hematologic test, weight change, and limb swelling were tracked.

**Results:**

Our results indicate that curcumin had a rather weak effect on arthritis progression in the Wistar rat model, microencapsulated curcumin effectively prevented the progression of arthritis – the disease stabilized after 10 days of supplementation. It also reduced the levels of immune cells (neutrophils and leukocytes), as well as pro-inflammatory cytokines – TNFα, IL-1, and IL-6, which levels were close to arthritis-free control. Other formulations of curcumin had lower or no effect on arthritis progression.

**Conclusion:**

Our study shows that the same concentrations of curcumin had a distinctly expressed positive anti-inflammatory effect depending on the form of its delivery. Specifically, we found that microencapsulated curcumin had the most promising effect for treatment.

**Graphical abstract:**

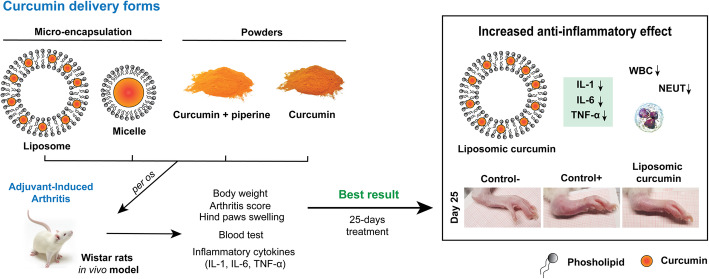

**Supplementary Information:**

The online version contains supplementary material available at 10.1186/s12906-021-03207-3.

## Background

Arthritis is a systemic disease characterized by inflammation of multiple joints affecting joint cartilage. Arthritis causes pain, stiffness, swelling of the joints, restricts the range of motion, decreases strength, and affects the quality of life. Approximately 3% of the worldwide population is affected by this disease [1]. It causes not only physical discomfort and pain but also places a person at an increased risk of work disability as arthritis usually starts in people aged between 30 and 40 years [1]. Currently, there is no known cure for arthritis. Treatment includes physical therapy, lifestyle changes and medications, mainly nonsteroidal anti-inflammatory drugs (NSAIDs). Although NSAIDs decrease both arthritis inflammation as well as pain, there is enough evidence that they increase the risk of gastrointestinal and cardiovascular complications, mainly due to the prolonged usage of large doses [2].

*Curcuma longa L.* rhizomes have been used for centuries in India and other south Asia countries as food, spice, as well as medicine for a wide variety of ailments, such as skin, pulmonary, gastrointestinal diseases, various aches, wounds, liver disorders [3]. In the past decades, curcumin, an active extract of turmeric, has gained much attention from the scientific community as a strong anti-inflammatory and antioxidant agent with a low incidence of side effects. Curcumin was shown to have potential against cancer, diabetes, Alzheimer’s, and cardiovascular diseases [3]. Multiple studies proved curcumin therapeutic effect against the progression of arthritis [4–7]. Arthritis has a strong inflammatory background. The main cytokines involved in the inflammatory cascade of arthritis are tumour necrosis factor-α (TNF-α), interleukin (IL)-6, and IL-1 [8]. Studies show that curcumin inhibits the production and activity of main inflammation-associated markers, such as TNF-α, nuclear factor-κB (NF-κB), cyclooxygenase-2 (COX-2), mammalian target of rapamycin (mTOR), interleukins. This effect enables curcumin to reduce inflammation in diseases like arthritis, psoriasis, inflammatory bowel disease, and asthma [9].

Curcumin extract is composed of three curcuminoids at different proportions – curcumin (~ 77%), demethoxycurcumin (17–18%), and bisdemethoxycurcumin (3–5%) [10]. When administered orally, curcumin is metabolized into curcumin glucuronide and curcumin sulfonate by the liver during phase II reactions. Glucuronidation and sulfation make curcumin more water-soluble, but decrease its effectiveness and accelerate curcumin removal via urine [11, 12]. Curcumin has a hydrophobic nature, which lowers its absorption and bioavailability [13]. Clinical studies show that pure curcumin extract is absorbed poorly – doses up to 8000–12,000 mg/day were traditionally given to volunteers, and only minimal amounts were found in their blood plasma [14]. Multiple strategies increasing the bioavailability of curcumin were proposed. Piperine, extracted from black peppers, is one of the most popular natural bioavailability enhancers [15]. There are two ways how piperine can enhance the bioavailability of curcumin. The first way is by increasing curcumin’s absorption by stimulating biliary excretion [16]. Curcumin is a lipid-soluble molecule, thus by increasing biliary excretion, it enhances lipid absorption, together with dissolved curcumins. The other mechanism of action is that piperine can inhibit the metabolism of curcumin [15, 17, 18]. There are many studies which show how piperine impacts the concentration of various drugs in serum by inhibiting proteins involved in active metabolism, for example, piperine could be a potent inhibitor of cytochrome P450 which is involved in warfarin – anticoagulant metabolism [17, 19–21]. Other innovative forms include curcumin nanoemulsion, micellar curcumin, cyclodextrin curcumin, which also increases curcumin bioavailability in both animal and human subjects [15, 22, 23].

Microencapsulated curcumin contains microsized vesicles dispersed in water. Microcapsules are composed of a phospholipid bilayer, made from natural ingredients, and can incorporate hydrophilic, hydrophobic, and amphiphilic molecules. Due to the similarity of phospholipid bilayer to cell membranes, microencapsulated nutrients are absorbed a few times better than standard oral supplements, as supported by both pre-clinical and clinical studies [24–26]. Lecithin-encapsulated curcumin fed to rats, increased the bioavailability of curcumin by up to 5 times. Plasma antioxidant activity was also significantly higher due to curcumin encapsulation in comparison to other oral forms [27]. Clinical studies confirm in vivo results. It was shown that curcumin and lecithin mixture increase curcuminoids in the blood plasma of healthy volunteers 29-fold higher than standard curcumin supplementation, even at a lower dose. Moreover, the maximum concentration of curcumin in blood plasma was reached more than 2 times faster with encapsulated curcumin than standard formulation [28].

In summary, the concentration of curcumin in oral supplements is not the only parameter impacting its effect. It is important to evaluate which form of curcumin preparation or mixture with other compounds has the biggest impact on its bioavailability and anti-inflammatory effect. This study aimed to evaluate the impact of different curcumin preparations in alleviating arthritis symptoms and inflammatory background.

## Methods

### Test substances

Different curcumin preparations with the provided code names were prepared at the pharmaceutical company Valentis and kindly donated for investigation. Liposomal curcumin (LIPO) syrup was composed of 34 mg/ml curcumin (microencapsulated in liposomes) and soya lecithin (phosphatidylcholine). This syrup is produced by Valentis and sold under the SmartHit trademark. Micellar curcumin (MIC) syrup was composed of polysorbate 80 (Thermo Fisher Scientific, MI, USA) and 63 mg/ml curcumin. Piperin containing curcumin (PIP) capsules were filled with powder, which consisted of 200 mg curcumin and 5 mg piperin. Basic curcumin (BAS) capsules contained 200 mg of curcumin extract powder.

### Animals

Thirty-two healthy Wistar rats of the same DOB (8-week-old, males 252 ± 12 g, females 184 ± 11 g; Vilnius University; Lithuania; Vet. Approval No LT 59–13-001, LT 60–13-001, LT 61–13-004) was used for the experiment. Before the start of the experiment, all animals were weighed and pilot blood samples (0 days before immunization) were analyzed to ensure that there are no differences in these parameters. Then according to pre-test results, the animals were randomly divided into 6 groups, thus ensuring that the median of each animal group was a similar prior experiment. Each study group consisted of 6 animals (3 males and 3 females), the negative (healthy animals), control (Control-), and positive (untreated) control (Control+) groups included 4 animals (2 males and 2 females) each. Experimental groups were formed using the resource equation method, a minimum number of animals was needed to calculate the statistical reliability of the data. For the experiment, each rat was housed in an individual standard rat cage (Tecniplast, dimensions of caging L x W x H, 480 × 375 × 210 mm, floor area 1500 cm^2^). Cages were filled with aspen chip bedding substrate (Tapvei) and had an enriched environment. Animals were supervised daily and maintained under standard controlled conditions: temperature + 22 ± 1 °C, humidity 55 ± 3%, and a 12 h light/12 h dark cycle with lights on at 7:00 a.m. and off at 7:00 p.m. During the experiment, rats were weighed once a week before procedures. They were fed with standard commercial rodent feed JE-83004920 (Joniskio grudai, Ltd., Lithuania) and had access to water ad libitum. All experimental procedures conformed to Directive 2010/63/EU requirements and were approved by the Lithuanian State Food and Veterinary Service (approval number G2–47, 30/06/2016).

### Adjuvant-induced arthritis (AIA)

Experimental arthritis in rats was induced at day 0, according to Chondrex, Inc. protocol *Protocol for Adjuvant-Induced Arthritis (AIA) in Rats* (2017). 100 μl of Complete Freund’s Adjuvant (CFA) (Chondrex, Redmond, WA, USA) containing 10 mg/ml of heat-killed *Mycobacterium tuberculosis* was injected subcutaneously to each animal at the base of the tail. According to the manufacturer, severe arthritis appears between days 12 to 14 and often persists until days 20 to 25. An additional table shows the experimental design (see Additional file 1).

### AIA – evaluation and treatment

The intensity of rats limb inflammation was evaluated by measuring the width of the paw joint. Measurements were made using a digital caliper on days 0, 6, 12, 15, 19, and 25, data is presented in mm. On the same days, the pictures of the hind paws were taken. In addition to these measurements, the daily intensity of the inflammatory process of each animal was scored (starting on day 6 after CFA injection) according to Chondrex, Inc. recommendations. The arthritis score equivalents were as follows: 0 points – no changes in limbs and animal health, 1 – paw becomes red, 2 – swollen paw, 3 – swollen paw and joint, also joints of fingers, 4 –whole paw swollen, swollen paw and finger joints, the animal has difficulty moving the limb, noticeable worsening of general health. Treatment with different curcumin preparations was started at day 6 (after the first AIA symptoms were observed) and was continued until day 25. All curcumin containing test substances were prepared before every administration to a final curcumin concentration of 170 mg/kg per dose. This concentration was chosen according to the literature [4, 29–31]. Syrups (LIPO and MIC) were not diluted, powders (PIP and BAS) were prepared in pure olive oil. All preparations were administered per os once daily at 8:00 a.m. To reduce the possibility of subjective assessment, all curcumin preparations before the experiment were coded by an independent investigator using numbers 1, 2, 3, and 4, the same coding was provided for animal cages. All paw swelling assessments were performed at least by two people, one measured parameters blindly (not knowing from which group the animal was brought to him by the second investigator). To maintain animal welfare, animals were brought to the test laboratory before the procedures and left for 30 min to familiarize themselves with the environment. While procedures were performed on an individual animal, the other animals were kept out of sight.

### Blood sampling and analysis

Blood samples were collected from the non-anaesthetized rat tail vein with a 22 G butterfly needle in a 20 μl ethylenediaminetetraacetic acid (EDTA) covered capillary and immediately analyzed with a veterinary heamatology analyzer Exigo EOS (Jainam Biomedical). Samples were analyzed on day 0 (before immunization) as a control reference point for each animal inflammatory process. Later blood parameters were measured at days 6, 12, 19, and 25 after CFA injection. White blood cells (WBC) (× 10^9^/L), neutrophils (NEUT) (× 10^9^/L), red blood cells (RBC) (× 10^9^/L) and haemoglobin (HGB) (g/dL) levels were evaluated in all adjuvant-induced arthritis and control rats groups.

### Cytokine determination

Serum samples were collected at the last experiment day (day 25; after 20 consecutive days of treatment). Animals were euthanized according to *AVMA Guidelines for the Euthanasia of Animals: 2020 Edition*. Rats one by one were placed in a transparent chamber where a flow of 8.0 L/min of medical CO_2_ gas (Elme Messer) was introduced for at least 5 min. After assessing the signs of death, decapitation was performed and 1 mL of blood from the heart and chest cavity was collected in clean tubes. After 3 h at RT, blood serum was isolated by 15 min centrifugation at 1500 rpm at + 4 °C using a centrifuge Z 326 K (HERMLE Labortechnik GmbH). Separated serum (500–900 μL) was transferred in clean tubes and kept in − 80 °C Forma 900 Series freezer (Thermo Fisher Scientific, MI, USA) until the beginning of the assessment. Inflammatory cytokines in rat’s serum were determined by enzyme-linked immunosorbent assay (ELISA) using commercial kits specific for rat IL-1 (BMS630), IL-6 (BMS625), and TNF-α (BMS622) (Invitrogen, Thermo Fisher Scientific, MI, USA). All samples and standards were assayed in duplicate; absorbance was measured at 450 nm using Varioskan Flash spectrophotometer (Thermo Fisher Scientific, MI, USA). The average optical density of duplicates was used for a standard curve creation and cytokines concentration determination. All procedures and data analyses were performed according to the manufacturer’s instructions.

### Statistical analysis

Data analysis was performed using GraphPad Prism version 6.01 for Windows (GraphPad Software, La Jolla California USA). Differences between groups of animals (weight change, hind paws swelling, and blood parameters) at the respective study days were assessed by one-way ANOVA followed by the Tukey LSD post hoc test. Data were considered statistically significant when *p* < 0.05. The data is shown as the groups’ mean points and connecting lines with ± standard deviation (SD) or scatter plot and presented as a median with interquartile range (IQR). ELISA test results of serum cytokine concentrations in the samples were calculated from standard curves plotted against a 5 parameter curve fit. Concentrations were expressed as pg/ml for every single animal; confidence interval (CI) 95% was calculated according to the designed model.

## Results

**Fig. 1:**
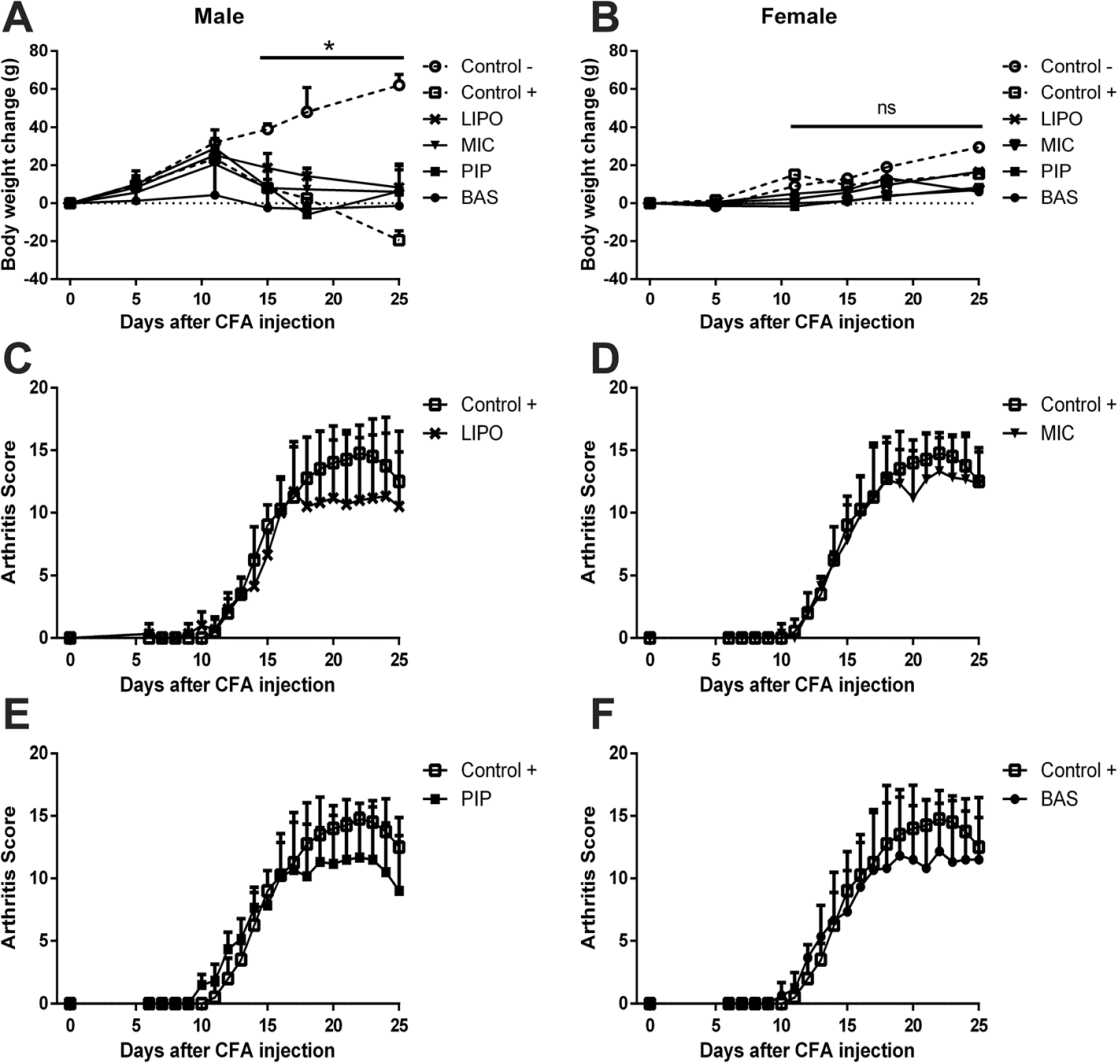
Body weight and arthritis score evaluation of Wistar rats after AIA initiation. Data are shown as mean ± SD. **a-b** Body weight change in rats at 0, 5, 11, 15, 18, and 25 days after CFA injection. *(*p* < 0.05) - denotes statistically significant differences compared with Control– group, ns - not significant. Detail statistical analysis of the male group (see Additional file 2). Data table presented in Additional file 3. **c-f** Daily arthritis score measurements of rat paws over 25 days. Comparison of Control+ and Control- groups arthritis score (see Additional file 4). Data table presented in Additional file 5

The weight change of all Control- (healthy animals) rats increased consistently during the study, while positive control Control+ (untreated animals) males started to lose weight from day 11 after adjuvant-induced arthritis (AIA) initiation. This effect was not evident in Control+ rat females; unlike males, their weight remained almost unchanged (Fig. 1.a-b). All males treated with curcumin, irrespective of the used preparation, showed a tendency to lose weight. In contrast, in females, no weight loss was observed, which indicates that all curcumin delivery forms had a small positive effect on animal weight compared with the Control+ group, although no statistically significant results were determined.

The swelling of animal hind paws is the best indicator of experimental arthritis. Consistent evaluation of arthritis score after AIA initiation showed that treatment with different curcumin preparations delayed the onset of arthritis symptoms; however, due to the data scattering, no significant effects were observed (Fig. 1.c-f). Analysis of arthritis scores indicated the postpone effect of arthritis progression in the liposomal curcumin (LIPO), piperin containing curcumin (PIP), and basic curcumin (BAS) groups. Following the initial administration of these curcumin preparations, rat paws were less swollen, whereas micellar curcumin (MIC) had almost no effect compared to the non-treated Control+ group. From day 17, we observed that curcumin began to show a positive effect on all treated animals groups, including the MIC curcumin preparation received group. However, as at the beginning of arthritis, micellar curcumin was the least effective in reducing symptoms of the disease.

**Fig. 2:**
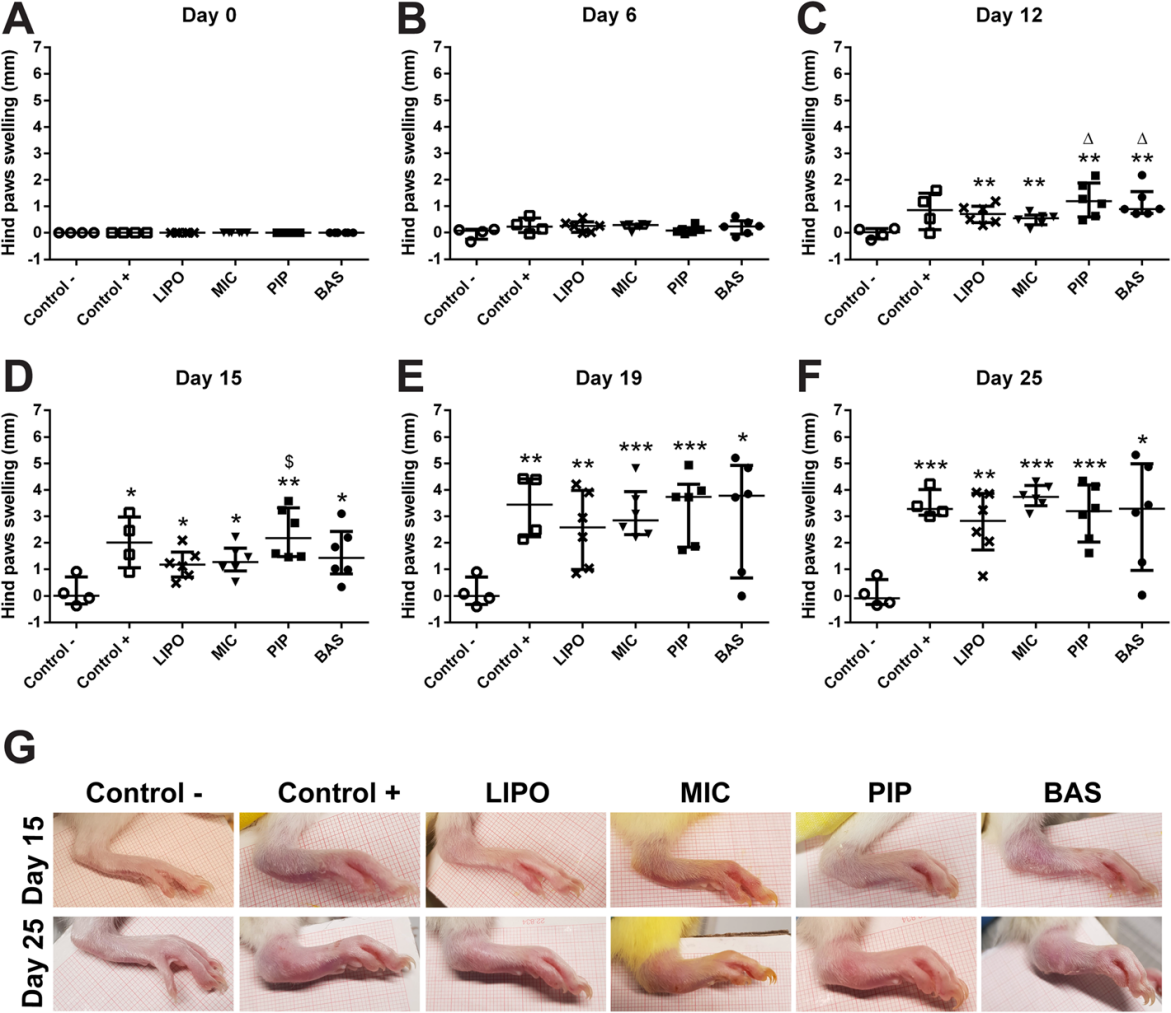
Hind paws swelling in Wistar rats at 0, 6, 12, 15, 19, and 25 days after AIA initiation. Data are shown as median with IQR. **a-f** Hind paws swelling measurements, * denotes statistically significant differences Control– vs group - *(*p* < 0.05), **(*p* < 0.01), ***(*p* < 0.001), while Δ(*p* < 0.05) denotes statistically significant differences compared to MIC group, and $(*p* < 0.05) denotes statistically significant differences compared to LIPO group. **g** Pictures of swelled hind paws 15 and 25 days after CFA injection. All treatment period (see Additional file 6)

During arthritis progression, paw swelling was also measured (Fig. 2.a-f). For the first 15 days, LIPO and MIC groups showed the highest efficacy in preventing the swelling of the paws compared with other groups (Fig. 2.a-d). PIP-administered rats demonstrated the same paws swelling tendency as the Control+ group. However, after 15 days, no anti-swelling effect was observed in either group. It is clear that the disease was far too advanced, and the anti-inflammatory effects of the tested curcumin products were no longer sufficient to control the disease. Photographs of the hind paws (Fig. 2.g) confirm these results. After 15 days of treatment, LIPO and MIC groups were visually comparable to Control-, while after 25 days of treatment, the paws of all treated groups showed no difference from the Control+ group animals’ paws.

**Fig. 3:**
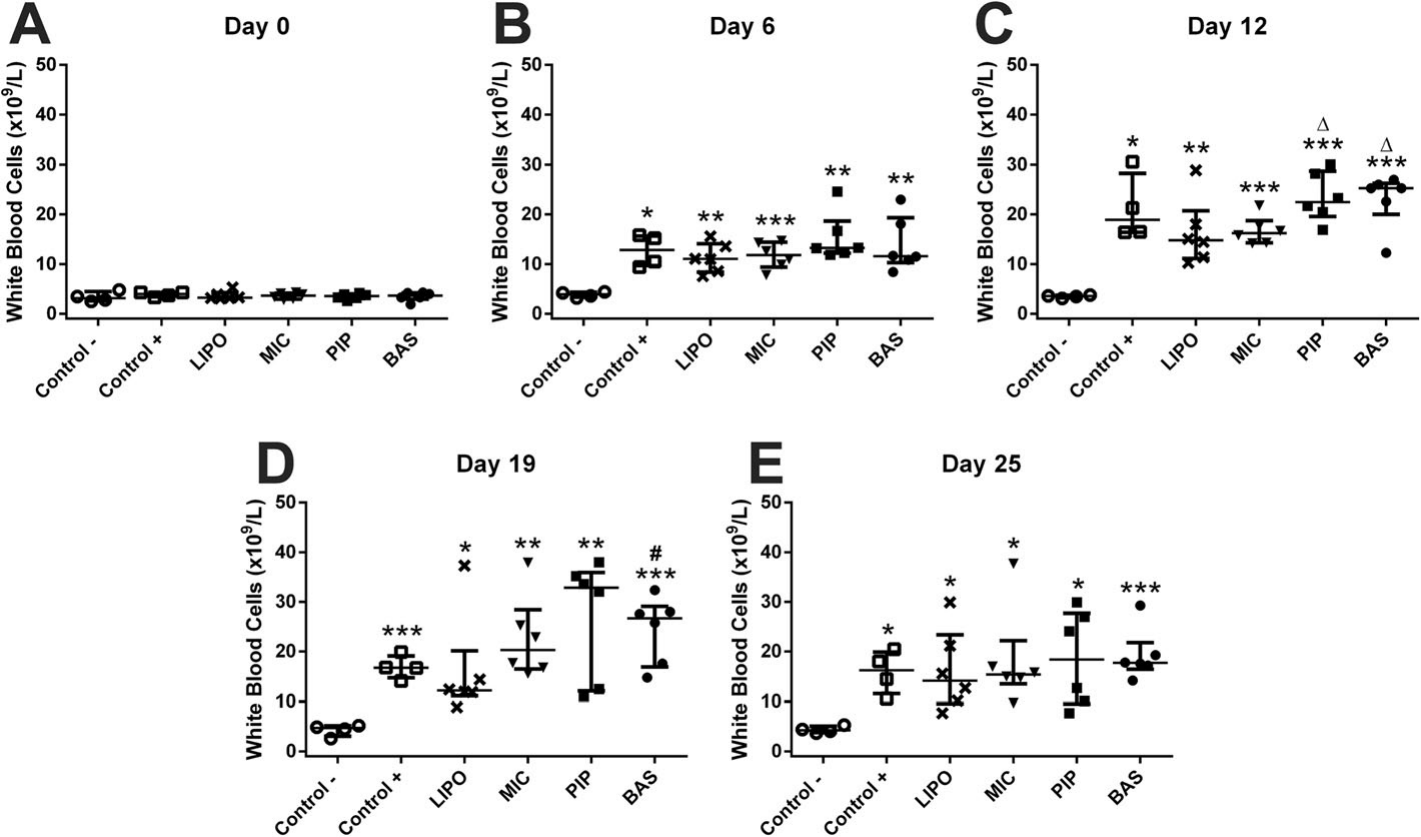
Levels of white blood cells in Wistar rats at 0, 6, 12, 19, and 25 days after AIA initiation. Data are shown as median with IQR. * denotes statistically significant differences Control– vs group - *(*p* < 0.05), **(*p* < 0.01), ***(*p* < 0.001), while Δ(*p* < 0.05) denotes statistically significant differences compared to MIC group and #(*p* < 0.05) denotes statistically significant differences compared to Control+ group

Blood parameters measurements showed an increase in total white blood cell (WBC) concentration in all experimental groups 6 days after AIA initiation (before treatment) (Fig. 3.a-e). Differences in WBC levels were observed upon the beginning of the different curcumin preparations administration (after 6 days). LIPO and MIC were the most effective in suppressing WBC increase compared with other curcumin preparations. However, there were no significant differences compared with Control+ group. Moreover, in PIP and BAS groups, WBC levels exceeded the values of the Control+ group. Twenty-five days after complete Freund’s adjuvant (CFA) injection, the concentrations of WBC in all arthritis groups were almost equal regardless of what curcumin preparation was administered.

**Fig. 4:**
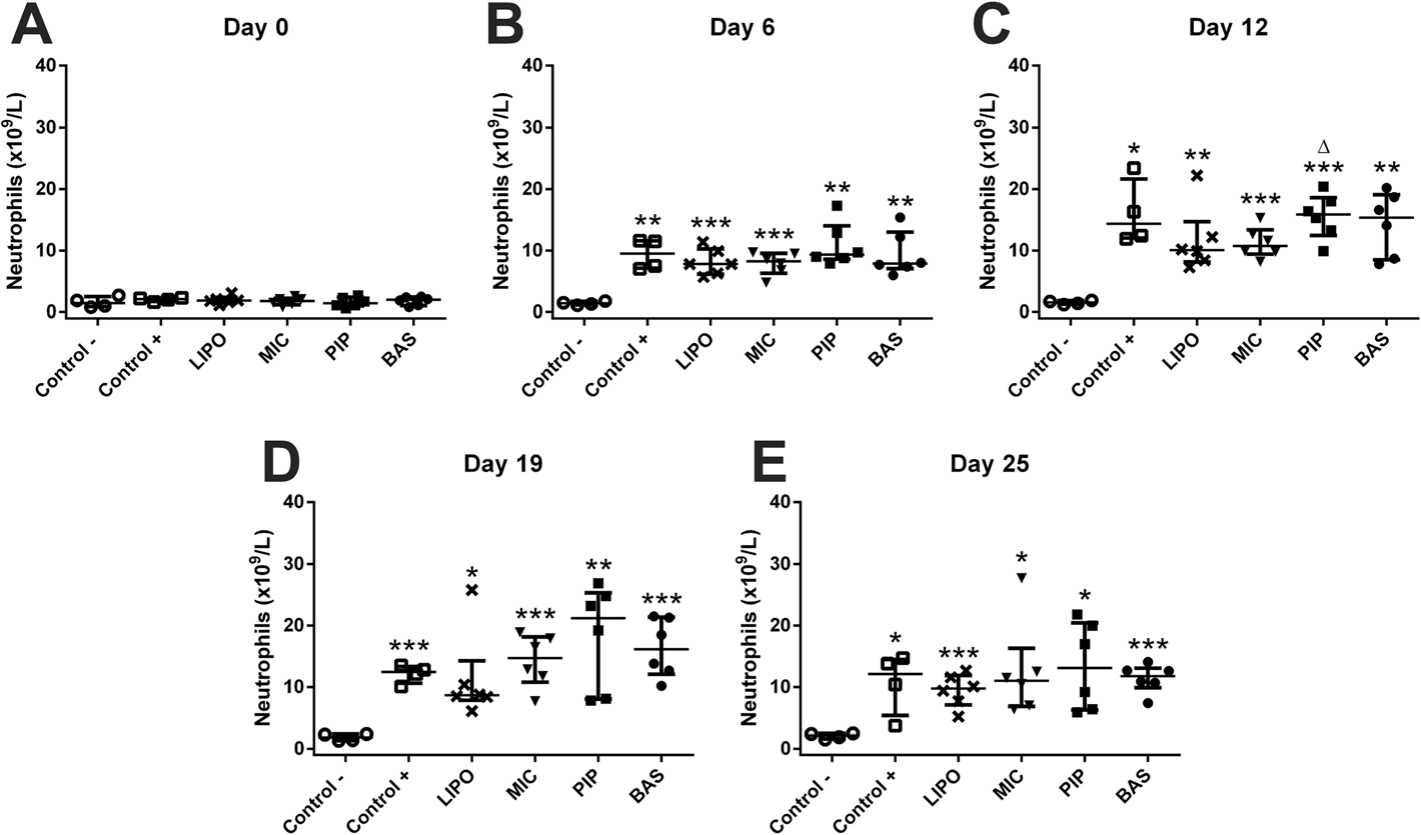
Levels of neutrophils in Wistar rats at 0, 6, 12, 19, and 25 days after AIA initiation. Data are shown as median with IQR. * denotes statistically significant differences Control– vs group - *(*p* < 0.05), **(*p* < 0.01), ***(*p* < 0.001) and Δ(*p* < 0.05) denotes statistically significant differences compared to MIC group. Comparison of neutrophil levels in separate groups during the experiment (see Additional file 7)

A similar effect was also observed in neutrophil (NEUT) levels (Fig. 4.a-e). LIPO and MIC curcumin syrups were able to stabilize NEUT levels; though, they remained elevated throughout the experiment. As well as WBC levels, rats administered with PIP and BAS preparations tended to have more elevated levels of neutrophils than untreated Control+ group animals. As we see in the treatment dynamics of the curcumin preparations used, only LIPO curcumin prevented a further increase of neutrophils (see Additional file 7).

**Fig. 5:**
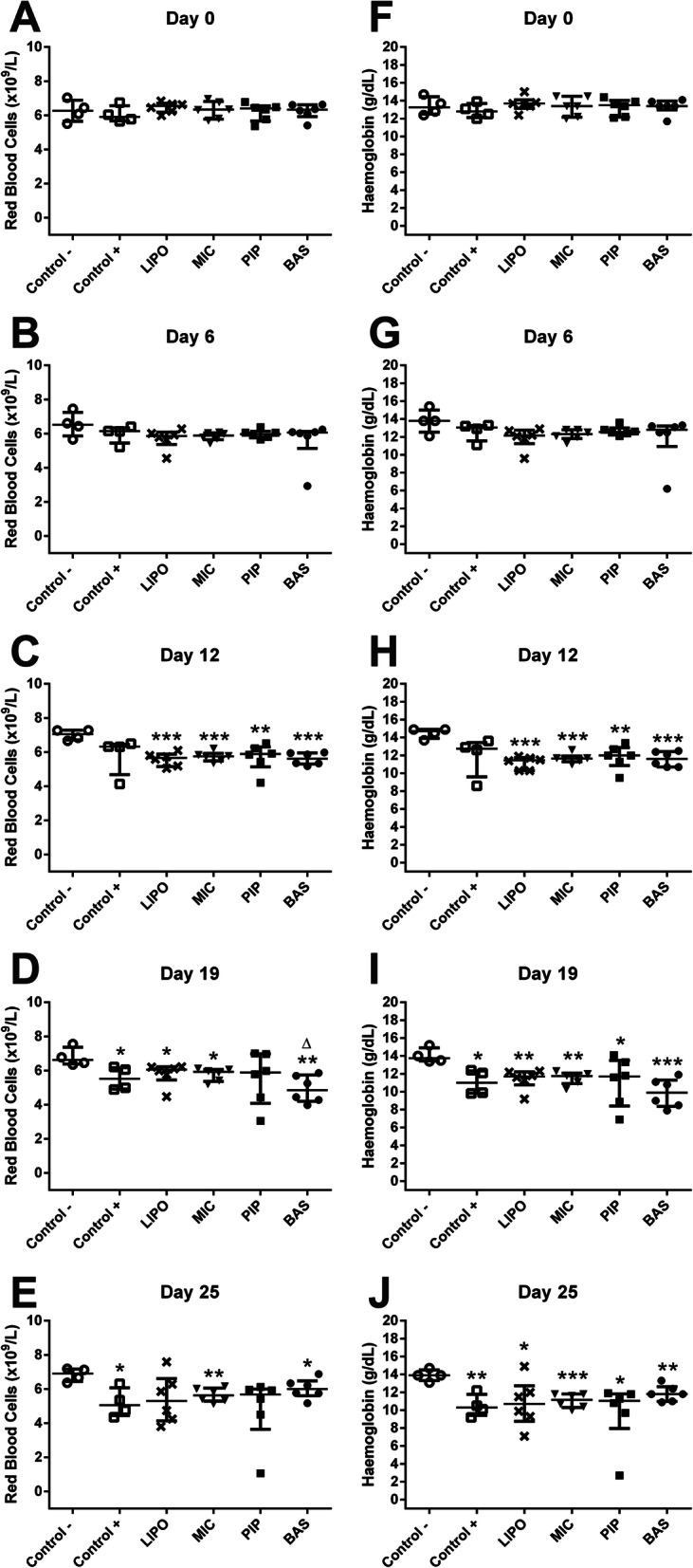
Levels of red blood cells (**a-e**) and haemoglobin (**f-g**) in Wistar rats at 0, 6, 12, 19, and 25 days after AIA initiation. Data are shown as median with IQR. * denotes statistically significant differences Control– vs group - *(*p* < 0.05), **(*p* < 0.01), ***(*p* < 0.001), and Δ(*p* < 0.05) denotes statistically significant differences compared to MIC group

Red blood cells (RBC) levels decreased as arthritis progressed (Fig. 5.a-e). None of the curcumin preparations used was able to elevate RBC levels back to the Control- group level. During later experiment time points (at days 19 and 25), the results were more dispersed, effects of curcumin depended on the individual rat. The same results were observed with haemoglobin (HGB) (Fig. 5.f-j). As arthritis progressed, RBC levels and HGB concentration decreased, and no positive impact of tested curcumin preparations was observed.

In this study, cytokine IL-1, IL-6, and TNF-α concentrations were measured at the end of the experiment. Blood was collected on the 25th day of the experiment, and cytokine concentrations were evaluated by ELISA test. Cytokine levels in animals were highly variable (Table 1). For this reason, the results for each individual were presented separately. Such presentation of data shows a general trend of how different curcumin preparations impact IL-1, IL-6, and TNF-α expression in rats. In the healthy Control- group, none of the tested cytokines were detected (concentrations were < 31.3 pg/mL – lower detection limit of ELISA). Thus, no inflammatory processes were present in this animal group. At 25th day, in the blood of untreated Control+ group animals IL-1 ranged from 33.8 to 196.4 pg/mL, IL-6 from < 31.3 to 205.7 pg/mL and TNF-α from < 39.1 to 736.6 pg/mL. The inflammation was the most pronounced in this experimental group. LIPO curcumin-treated group concentrations of all tested cytokines in all animals were reduced to control levels (less than the detection limit < 31.3 pg/mL) except one animal with 37.9 pg/mL concentration of IL-1 and 81.1 pg/mL of TNF-α. The effect of MIC varied; in some animals, the levels of the inflammatory cytokines reached Control- group parameters but remained elevated in the rest. Specifically, from < 31.3 to 193.6 pg/mL for IL-1, from < 31.3 to 212.7 pg/mL for IL-6, and from < 39.1 to 618.4 pg/mL for TNF-α. PIP curcumin reduced the levels of all cytokines. Only two rats had increased levels of IL-1 (37.7 and 91.5 pg/mL), and only one had increased concentration of IL-6 (96.9 pg/mL), while TNF-α concentrations in this group did not differ from those of healthy animals. IL-1 concentrations ranged from < 31.3 to 127.1 pg/mL and IL-6 – from < 31.3 to 149.5 pg/mL in animals treated with BAS curcumin, while the increased level of TNF-α was observed in one animal (107.3 pg/mL).

**Table 1:** Levels of cytokines IL-1, IL-6, and TNF-α in Wistar rats at 25 day after AIA initiation

Group	Sex	IL-1, pg/ml	95% CI	IL-6, pg/ml	95% CI	TNF-α, pg/ml	95% CI
Control -	Male	< 31.3	0 - < 31.3	< 31.3	0 - < 31.3	< 39.1	0 - < 39.1
	Male	< 31.3	0 - < 31.3	< 31.3	0 - < 31.3	< 39.1	0 - < 39.1
	Female	< 31.3	0–31.3	< 31.3	0 - < 31.3	< 39.1	0 - < 39.1
	Female	< 31.3	< 31.3–50.1	< 31.3	< 31.3–56.9	< 39.1	0 - < 39.1
Control +	Male	**33.8**	< 31.3–76.0	< 31.3	< 31.3–68.1	< 39.1	< 39.1–67.1
	Male	**54.0**	< 31.3–98.0	**36.1**	< 31.3–73.9	< 39.1	< 39.1–66.9
	Female	**196.4**	155.6–235,4	**205.7**	170.8–239.4	**736.6**	767.5–190.5
	Female	**51.5**	< 31.3–95.0	**63.7**	< 31.3–99.5	**226.1**	190.5–261.4
LIPO	Male	< 31.3	< 31.3–39.9	< 31.3	< 31.3–35.4	< 39.1	0 - < 39.1
	Male	< 31.3	< 31.3–54.0	< 31.3	< 31.3–43.8	< 39.1	0 - < 39.1
	Male	< 31.3	< 31.3–39.7	< 31.3	0 - < 31.3	< 39.1	0 - < 39.1
	Female	< 31.3	0 - < 31.3	< 31.3	0 - < 31.3	< 39.1	0 - < 39.1
	Female	< 31.3	0 - < 31.3	< 31.3	0 - < 31.3	< 39.1	0 - < 39.1
	Female	**37.9**	< 31.3–80.5	< 31.3	< 31.3–61.7	**81.1**	120.8
MIC	Male	**41.4**	< 31.3–83.0	**33.4**	< 31.4–72.1	**80.9**	41.7–118.0
	Male	**114.9**	73.4–155.0	**127.7**	91.5–162.5	< 39.1	0 - < 39.1
	Male	< 31.3	0 - < 31.3	< 31.3	0 - < 31.3	**202.4**	165.0–238.6
	Female	< 31.3	0 - < 31.3	< 31.3	0 - < 31.3	< 39.1	0 - < 39.1
	Female	**193.6**	152.7–234.2	**212.7**	177.7–247.1	**618.4**	585.9–649.4
	Female	< 31.3	< 31.3–57.4	< 31.3	< 31.3–41.1	< 39.1	< 39.1–75.0
PIP	Male	**91.5**	49.6–134.2	**96.9**	58.6–132.6	< 39.1	< 39.1–74.0
	Male	< 31.3	0 - < 31.3	< 31.3	0 - < 31.3	< 39.1	0 - < 39.1
	Male	< 31.3	< 31.3–61.5	< 31.3	< 31.3–48.1	< 39.1	0 - < 39.1
	Female	< 31.3	0 - < 31.3	< 31.3	0 - < 31.3	< 39.1	0 - < 39.1
	Female	< 31.3	0 - < 31.3	< 31.3	0 - < 31.3	< 39.1	< 39.1–52.3
	Female	**37.7**	< 31.3–79.3	< 31.3	0 - < 31.3	< 39.1	0 - < 39.1
BAS	Male	**79.5**	37.2–120.5	**70.6**	< 31.3–107.3	< 39.1	< 39.1–73.2
	Male	< 31.3	< 31.3–62.2	< 31.3	< 31.3–50.0	< 39.1	0 - < 39.1
	Male	**127.1**	86.3–168.4	**149.5**	113.4–184.9	< 39.1	< 39.1–69.3
	Female	< 31.3	0 - < 31.3	< 31.3	0 - < 31.3	< 39.1	< 39.1–55.9
	Female	**83.5**	40.3–125.6	**104.0**	67.5–140.3	**107.3**	69.7–145.9
	Female	< 31.3	0 - < 31.3	< 31.3	< 31.1–34.1	< 39.1	0 - < 39.1

Data are shown as mean ± 95% confidence interval (CI). Concentrations (pg/ml) were calculated from standard curves plotted against a 5 parameter curve fit. CI 95% was calculated according to the designed model

## Discussion

Lately, medicine tries to move toward prevention and phytotherapy rather than treatment with anti-inflammatory drugs that are known to cause multiple side effects. It is known that inflammation is a causative agent in most chronic diseases; thus, natural extracts with immunosuppressive and anti-inflammatory properties are widely researched. Antioxidant molecules such as curcumin, resveratrol, quercetin can modulate cell signalling pathways associated with inflammatory arthritis [32]. At the molecular level, curcumin interacts with more than 30 different proteins [33]. Curcumin is known to inhibit one of the main transcription factors in inflammatory diseases – NF-κB. Inhibition of NF-κB explains curcumin anti-inflammatory and immunosuppressive activity [34].

Despite the positive effect of curcumin, its low bioavailability remains one of the major issues. Given as a pure extract, curcumin is quite unstable, poorly soluble in water, and quickly metabolized by the liver. Thus, scientists are constantly searching for a method to enhance its bioavailability. Piperine enhances the solubility of lipid-soluble compounds by stimulating bile secretion, increases membrane permeability and blood supply at the site of absorption, also inhibits enzymes responsible for curcumin metabolism [35]. As an alternative, the food supplement industry adapted microencapsulation technology that originated in oncology. First studies on nutraceutical encapsulation in liposomes began only less than two decades ago [36]. Microcapsules are sphere-shaped vesicles consisting of one or more phospholipid bilayers and encapsulated materials inside. Since the initial studies, microcapsules gained much attention in biochemistry, biophysics, pharmacology, and became a pharmaceutical phenomenon [37]. Microcapsules attracted much attention due to their biocompatibility, ability to incorporate hydrophilic, hydrophobic, and amphiphilic molecules, higher target specificity, and reduced risk of toxic side effects, especially for chemotherapeutics [26]. Microcapsules are produced from phospholipids isolated from soy, sunflower, or egg yolk lecithins. Phosphatidylcholine is considered the most versatile of all phospholipids for liposome formation [26]. Phospholipids are beneficial themselves – phosphatidylcholine is one of the main structural components of cell membranes and is used to rebuild damaged cells.

Microencapsulation increases the bioavailability of encapsulated compounds. In our previous study, the bioavailability of three different vehicles – microcapsules, micelles, and lipids – were compared. The absorption of microencapsulated vitamin D_3_ was 25% higher in comparison to the most commonly used oil-based supplements and twice higher than micellized vitamin D_3_. Further, the effect of microencapsulated vitamin D supplements was the longest and remained even after these supplements termination [38].

Our results demonstrated that after the symptomatic onset of arthritis, the daily administration of curcumin for 20 consecutive days may ease disease progression and reduce inflammatory background in rats. Adjuvant-induced arthritis is one of the most commonly used models of arthritis in rats due to shared similarities with human chronic inflammatory arthritis [39]. It is known that rats and humans share a majority of their biochemical properties at the genome level, which gives an important role for rats by considering them as a model organism for understanding human biology and diseases. However, despite an extremely small number of species-specific differences at the genome level, differences between humans and rats genes expression patterns in some cases can alter physiological responses to the same stimulus. To compare the effectiveness of lipophilic water-insoluble curcumin extract, we tested four different preparations of curcumin – basic, curcumin and piperine combination, micellar, and liposomal. None of the administered preparations affected the weight of the animals but showed differences in swelling of the hind paws. Liposomal and micellar curcumin statistically significantly slowed down the progression of arthritis. Despite a positive effect, at the end of the experiment, visual examination of hind paws showed no disparity between treatments with different curcumin preparations.

Complete blood test parameters not only shows inflammation due to induced arthritis but also provide important information about the general condition of the patient, reveals the intensity of the disease. Arthritis develops as an active inflammatory process, which manifests by increased total leukocyte levels and anaemia. Neutrophils are the main leukocytes, which number increases in this connective tissue disease and are critical to its progression. Neutrophils release cytotoxic and immunoregulatory molecules, cause oxidative stress through the production of reactive oxygen species, all these factors drive further development of arthritis [40]. In our study, an increase in leukocytes upon arthritis initiation was detected. Liposomal and micellar curcumin effectively decreased white blood cell levels, while curcumin with piperine and basic curcumin increased and even exceeded the values of the untreated control. Whereas the stabilization of neutrophil levels was detected in the liposomal curcumin-treated group. Cytokines involved in the pathogenesis of arthritis are classified into inflammatory and anti-inflammatory according to their function. Arthritis manifests with the elevation of pro-inflammatory cytokines such as IL-1, IL-6, and TNF-α. Although our used AIA rat model demonstrated a significant increase in IL-1, IL-6, and TNF-α levels, however, our study also had some limitations. The disease model we used showed that the development of joint arthritis differs slightly depending on the individual animal. For this reason, we could not directly evaluate the overall effect of curcumin preparations on pro-inflammatory cytokines comparing different groups of animals. Nevertheless, after treatment with different curcumin preparations, a decrease in cytokine production in rats was apparent. The results might be attributed to the anti-oxidative and neutrophil inhibitory activity of curcumin. Curcumin anti-oxidative effect and reduction of cytokines were also demonstrated in other studies, for example, Arora et al. evaluated the effectiveness of curcumin-loaded solid lipid nanoparticles for treating rheumatoid arthritis in rats. This study showed that the administration of curcumin reduced arthritis symptoms such as joint stiffness, hyperalgesia, oxidative stress, and TNF-α levels, improved mobility, expression of biochemical markers, and had the joint-protective potential [41]. In the recent study by Wang et al., bovine type II collagen-induced arthritis in rats was treated with curcumin. This treatment resulted in reduced joint swelling and the prevention of further disease progression. Cytokines TNF-α, IL-1β, IL-17, and transforming growth factor-β (TGF-β) levels were also significantly lowered after 10-days of treatment, and the effect was comparable with methotrexate, a drug used to treat rheumatoid arthritis [31]. Clinical studies confirm that curcumin is pharmacologically active not only in animal models but also in human subjects. According to a review of randomized clinical trials on curcumin in joint osteoarthritis, curcumin reduces pain and osteoarthritis index compared to placebo. Moreover, this meta-analysis concluded that there is no significant difference between curcumin and commercially available pain medicines such as ibuprofen, diclofenac, and glucosamine. This means what curcumin no only has similar analgesic properties but also is safer for long-term use [5].

Our results reveal that curcumin had a rather weak effect on arthritis progression in the Wistar rat model. LIPO curcumin preparation showed the most pronounced anti-arthritic effect, it managed to stabilize the disease progression after 10 consecutive days of its intake. LIPO curcumin also reduced the levels of immune cells (neutrophils and WBC), as well as pro-inflammatory cytokines – TNF-α, IL-1, and IL-6, which were close to healthy control. BAS and PIP curcumins also displayed a suppressory effect on arthritis progression, however, without affecting paw swelling. Moreover, BAS and PIP preparations had an immune reaction stimulating effect. Rats administered with BAS or PIP had increased levels of WBC and neutrophils, compared to the untreated positive control group. However, PIP displayed a more pronounced effect in lowering cytokine levels compared to BAS. MIC preparation effect on animal varied the most. MIC showed no effect in paw swelling but still had an impact on arthritis progression. As well as LIPO, MIC reduced the levels of WBC and neutrophils, conversely, it was not able to reduce cytokine concentrations, which shows ongoing inflammation in rats.

The overall positive effect of LIPO curcumin seen in rats might be attributed to an increased bioavailability of curcumin and the prevention of liver-driven first-pass metabolism, which decreases the serum concentration of curcumin and its effectiveness [42]. Previous studies confirm liposome-based curcumin efficacy to alleviate symptoms of arthritis, even in clinical cases. The curcumin-phosphatidylcholine complex was used for physiological functions of osteoarthritic patients improvement. Results obtained from a clinical trial showed an increase in patients distance walked, while reduced pain, stiffness, and inflammatory markers in organism helped to improve the overall life quality of patients. Also, the long-term tolerability of such curcumin preparation was evaluated and proved to be a safe alternative to NSAIDs in managing osteoarthritis symptoms [43].

## Conclusion

This study shows that curcumin has an anti-inflammatory effect and slows down the progression of arthritis in rats by reducing the levels of the main inflammatory cytokines and immune cells. Moreover, the intensity of this effect depended on curcumin preparation. Microencapsulated liposomal curcumin showed the most promising results. Despite the fact that our study does not show clearly expressed differences among tested curcumin preparations, our findings suggest that liposomal curcumin is a promising candidate for further arthritis treatment studies.

## Supplementary Information

**Additional file 1.** Experimental design.

**Additional file 2.** Statistical data table of mice weight change.

**Additional file 3. **Table of body weight changes in different experimental groups at 0, 5, 11, 15, 18, and 25 days after CFA injection. Animal weight changes are shown as average ± SD. *(*p* < 0.05) - denotes statistically significant differences compared with Control- group.

**Additional file 4. **Comparison of control groups arthritis score. Data are shown as mean ± SD. Marks *, #, Δ denotes statistically significant differences Control– vs Control+ − * (*p* < 0.05), # (*p* < 0.01) and Δ (*p* < 0.001).

**Additional file 5. **Table of arthritis score evaluation in rats during 25 days interval after AIA initiation. The arthritis score is shown as average ± SD. *(*p* < 0.05) denotes statistically significant differences Control– vs Control+.

**Additional file 6.** Hind paws swelling assessment during 25 days period after AIA initiation.

**Additional file 7. **Neutrophils parameters in groups. Comparison of neutrophils in each group during the experiment. Data are shown as median with IQR. * denotes statistically significant differences Control– vs group - *(*p* < 0.05), **(*p* < 0.01).

## Data Availability

The datasets used or analyzed during the current study are available from the corresponding author on reasonable request.

## Abbreviations

NSAIDs: Nonsteroidal anti-inflammatory drugs
TNF-α: Tumour necrosis factor-α
IL: Interleukin
NF-κB: Nuclear factor-κB
COX-2: Cyclooxygenase-2
mTOR: Mammalian target of rapamycin
LIPO: Liposomal curcumin
MIC: Micellar curcumin
PIP: Piperin containing curcumin
BAS: Basic curcumin
DOB: Date of birth
AIA: Adjuvant-induced arthritis
CFA: Complete Freund’s adjuvant
EDTA: Ethylenediaminetetraacetic acid
WBC: White blood cells
NEUT: Neutrophils
RBC: Red blood cells
HGB: Haemoglobin
RT: Room temperature
ELISA: Enzyme-linked immunosorbent assay
TGF-β: Transforming growth factor- β
