# Supplementary figures and images for: Anti-inflammatory effect of different curcumin preparations on adjuvant-induced arthritis in rats
Source: BMC Complement Med Ther. 2021 Jan 21;21:39. doi: 10.1186/s12906-021-03207-3 (PMC7819195; doi:10.1186/s12906-021-03207-3)

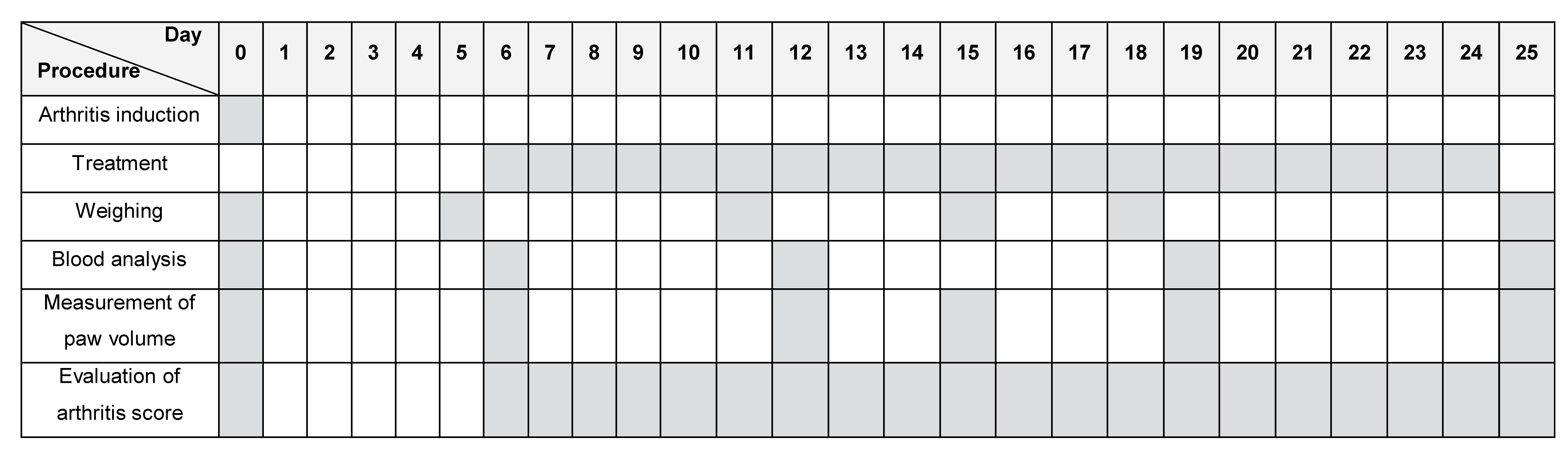

Supplement: Supplementary file 1 — Additional file 1. Experimental design. [file 12906_2021_3207_MOESM1_ESM.tif]

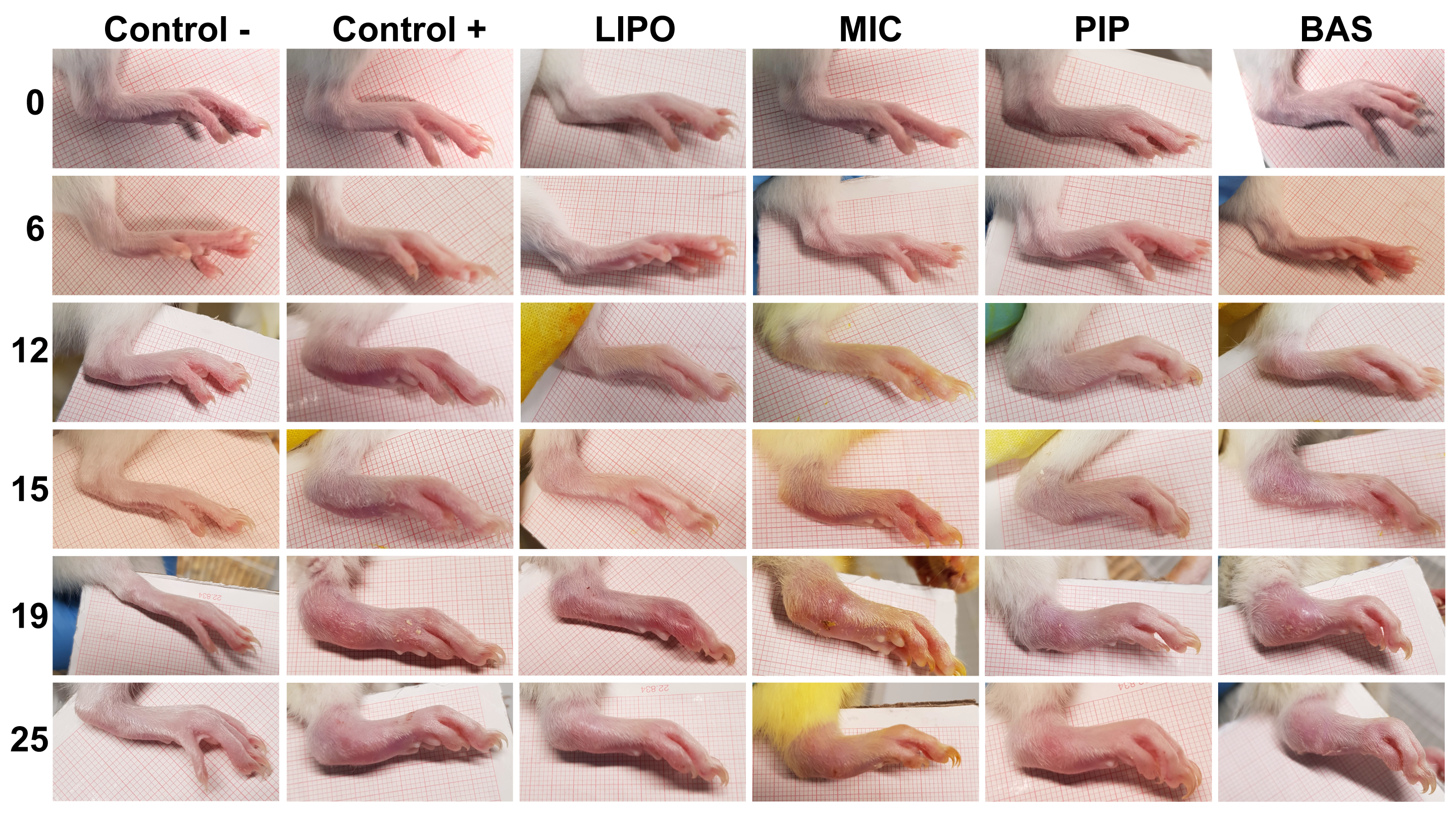

Supplement: Supplementary file 6 — Additional file 6. Hind paws swelling assessment during 25 days period after AIA initiation. [file 12906_2021_3207_MOESM6_ESM.tif]

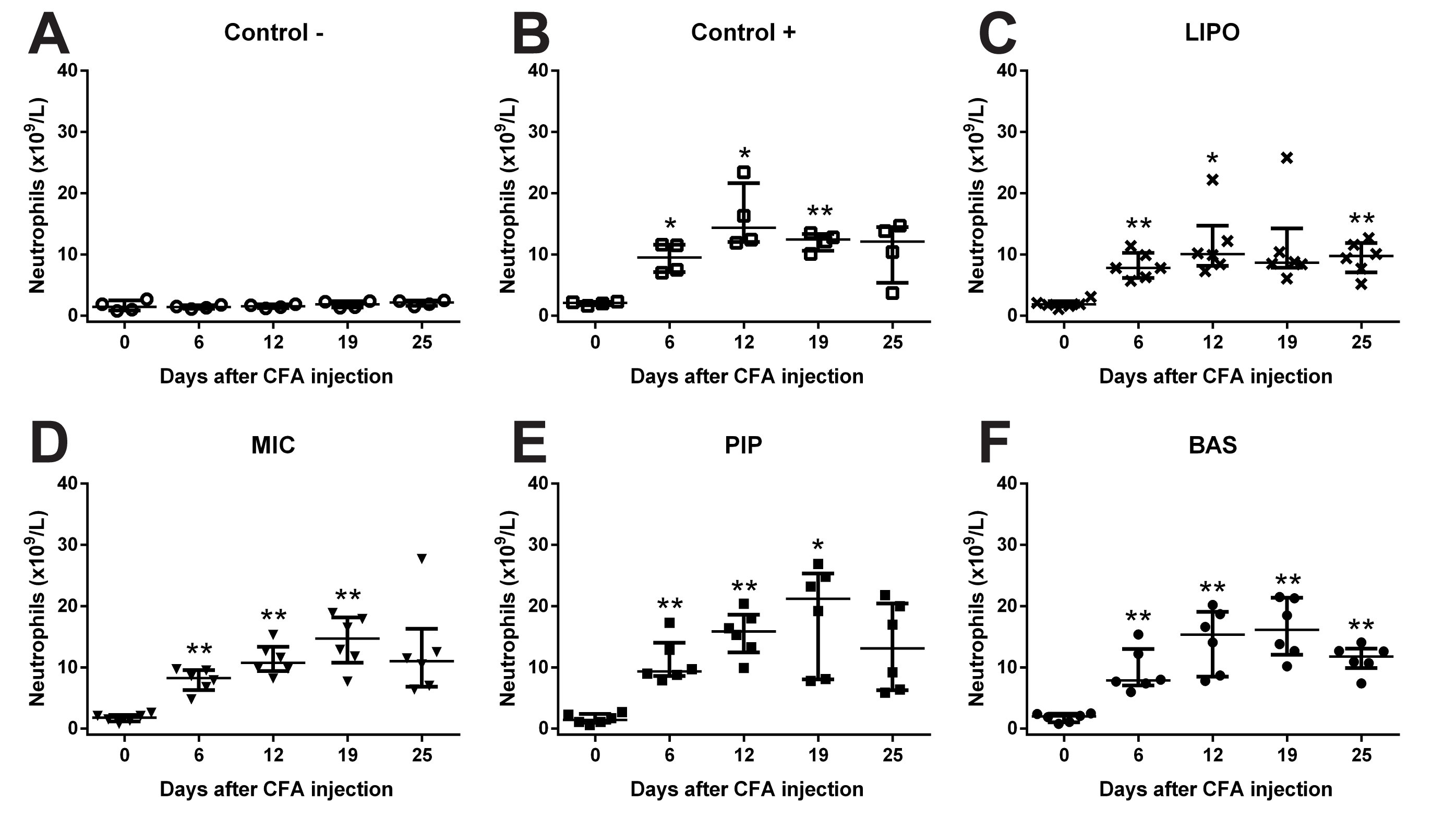

Supplement: Supplementary file 7 — Additional file 7. Neutrophils parameters in groups. Comparison of neutrophils in each group during the experiment. Data are shown as median with IQR. * denotes statistically significant differences Control– vs group - *(p < 0.05), **(p < 0.01). [file 12906_2021_3207_MOESM7_ESM.tif]
