# Supplementary material for: Anti-inflammatory effect of different curcumin preparations on adjuvant-induced arthritis in rats
Source: BMC Complement Med Ther. 2021 Jan 21;21:39. doi: 10.1186/s12906-021-03207-3 (PMC7819195; doi:10.1186/s12906-021-03207-3)
Supplement: Supplementary file 2 — Additional file 2. Statistical data table of mice weight change. [file 12906_2021_3207_MOESM2_ESM.docx]

**Additional file 2.** Statistical data table of mice weight change

| **One way ANOVA** | | | | | | **Tukey`s *post-hoc* multiple comparisons test** | | | | | |
| --- | --- | --- | --- | --- | --- | --- | --- | --- | --- | --- | --- |
| **Day 15** | | | | | | | | | | | |
|  | SS | df | MS | F | p | Group | Control+ | LIPO | MIC | PIP | BAS |
| Between groups | 2321 | 5 | 464.2 | 8.087 | 0.0027 | Control- | **0.0196** | 0.1118 | **0.0110** | **0.0126** | **0.0014** |
| Within group | 547.0 | 10 | 57.40 |  |  | Control+ |  | 0.6485 | >0.9999 | >0.9999 | 0.6753 |
|  |  |  |  |  |  | LIPO |  |  | 0.5469 | 0.6068 | 0.0563 |
|  |  |  |  |  |  | MIC |  |  |  | >0.9999 | 0.5767 |
|  |  |  |  |  |  | PIP |  |  |  |  | 0.5175 |
| **Day 18** | | | | | | | | | | | |
|  | SS | df | MS | F | p | Group | Control+ | LIPO | MIC | PIP | BAS |
| Between groups | 4326 | 5 | 865.1 | 15.79 | 0.0002 | Control- | **0.0011** | **0.0053** | **0.0013** | **0.0001** | **0.0002** |
| Within group | 547.8 | 10 | 54.78 |  |  | Control+ |  | 0.5321 | 0.9756 | 0.8001 | 0.9583 |
|  |  |  |  |  |  | LIPO |  |  | 0.8463 | 0.0590 | 0.1244 |
|  |  |  |  |  |  | MIC |  |  |  | 0.3132 | 0.5549 |
|  |  |  |  |  |  | PIP |  |  |  |  | 0.9952 |
| **Day 25** | | | | | | | | | | | |
|  | SS | df | MS | F | p | Group | Control+ | LIPO | MIC | PIP | BAS |
| Between groups | 7612 | 5 | 1522 | 14.01 | 0.0003 | Control- | **0.0002** | **0.0021** | **0.0016** | **0.0016** | **0.0006** |
| Within group | 1087 | 10 | 108.7 |  |  | Control+ |  | 0.1143 | 0.1636 | 0.1556 | 0.4489 |
|  |  |  |  |  |  | LIPO |  |  | 0.9997 | 0.9999 | 0.8560 |
|  |  |  |  |  |  | MIC |  |  |  | >0.9999 | 0.9476 |
|  |  |  |  |  |  | PIP |  |  |  |  | 0.9377 |
