# Supplementary material for: Anti-inflammatory effect of different curcumin preparations on adjuvant-induced arthritis in rats
Source: BMC Complement Med Ther. 2021 Jan 21;21:39. doi: 10.1186/s12906-021-03207-3 (PMC7819195; doi:10.1186/s12906-021-03207-3)
Supplement: Supplementary file 3 — Additional file 3. Table of body weight changes in different experimental groups at 0, 5, 11, 15, 18, and 25 days after CFA injection. Animal weight changes are shown as average ± SD. *(p < 0.05) - denotes statistically significant differences compared with Control- group. [file 12906_2021_3207_MOESM3_ESM.docx]

**Additional file 3.** Table of body weight changes in different experimental groups at 0, 5, 11, 15, 18, and 25 days after CFA injection

| **Day** |  |  |  |  |  |  |  |  |  |  |  |  |
| --- | --- | --- | --- | --- | --- | --- | --- | --- | --- | --- | --- | --- |
|  | **Control-** | | **Control+** | | **LIPO** | | **MIC** | | **PIP** | | **BAS** | |
|  | Males | Females | Males | Females | Males | Females | Males | Females | Males | Females | Males | Females |
| **5** | **10.00** ± 7.07 | **-1.50** ± 0.71 | **9.50** ± 4.95 | **1.50** ± 3.54 | **8.33** ± 3.05 | **0.33** ± 2.31 | **5.67** ± 7.37 | **-0.33** ± 1.15 | **10.00** ± 3.46 | **-1.33** ± 4.04 | **1.33** ± 6.66 | **0.67** ± 3.05 |
| **11** | **32.00** ± 1.41 | **9.00** ± 8.48 | **23.50** ± 10.61 | **15.00** ± 4.24 | **25.33** ± 13.32 | **2.33** ± 1.15 | **20.67** ± 13.20 | **0.00** ± 14.42 | **29.00** ± 2.00 | **-1.67** ± 5.69 | **4.33** ± 21.55 | **5.00** ± 3.46 |
| **15** | **39.00** ± 2.83 | **13.00** ± 1.41 | **8.00** ± 1.41* | **9.50** ± 6.36 | **18.67** ± 7.57 | **6.67** ± 2.08 | **8.00** ± 4.58* | **1.00** ± 1.00 | **8.67** ± 8.62* | **1.33** ± 6.43 | **-2.33** ± 11.37* | **7.00** ± 2.00 |
| **18** | **48.00** ± 12.73 | **19.00** ± 1.41 | **2.50** ± 0.71* | **11.50** ± 10.61 | **14.33** ± 4.04* | **9.67** ± 2.52 | **7.33** ± 11.24* | **4.00** ± 5.00 | **-6.00** ± 4.36* | **3.67** ± 5.77 | **-3.00** ± 5.57* | **13.33** ± 5.86 |
| **25** | **25.00** ± 5.66 | **29.50** ± 2.12 | **-19.50** ± 4.95* | **15.5** ± 10.61 | **8.33** ± 9.29* | **16.67** ± 5.51 | **6.00** ± 13.86* | **7.00** ± 8.00 | **6.33** ± 14.29* | **8.33** ± 2.08 | **-1.33** ± 5.69* | **6.00** ± 22.91 |
|  |  |  |  |  |  |  |  |  |  |  |  |  |

Animal weight changes are shown as average ± SD. *(p<0.05) - denotes statistically significant differences compared with Control- group.
