# Supplementary material for: Anti-inflammatory effect of different curcumin preparations on adjuvant-induced arthritis in rats
Source: BMC Complement Med Ther. 2021 Jan 21;21:39. doi: 10.1186/s12906-021-03207-3 (PMC7819195; doi:10.1186/s12906-021-03207-3)
Supplement: Supplementary file 5 — Additional file 5. Table of arthritis score evaluation in rats during 25 days interval after AIA initiation. The arthritis score is shown as average ± SD. *(p < 0.05) denotes statistically significant differences Control– vs Control+. [file 12906_2021_3207_MOESM5_ESM.docx]

**Additional file 5.** Table of arthritis score evaluation in rats during 25 days interval after AIA initiation

| **Day** |  |  |  |  |  |  |
| --- | --- | --- | --- | --- | --- | --- |
|  | **Control-** | **Control+** | **LIPO** | **MIC** | **PIP** | **BAS** |
| **0** | 0.00 ± 0.00 | 0.00 ± 0.00 | 0.00 ± 0.00 | 0.00 ± 0.00 | 0.00 ± 0.00 | 0.00 ± 0.00 |
| **6** | 0.00 ± 0.00 | 0.00 ± 0.00 | **0.33** ± 0.82 | 0.00 ± 0.00 | 0.00 ± 0.00 | 0.00 ± 0.00 |
| **7** | 0.00 ± 0.00 | 0.00 ± 0.00 | 0.00 ± 0.00 | 0.00 ± 0.00 | 0.00 ± 0.00 | 0.00 ± 0.00 |
| **8** | 0.00 ± 0.00 | 0.00 ± 0.00 | 0.00 ± 0.00 | 0.00 ± 0.00 | 0.00 ± 0.00 | 0.00 ± 0.00 |
| **9** | 0.00 ± 0.00 | 0.00 ± 0.00 | **0.33** ± 0.82 | 0.00 ± 0.00 | 0.00 ± 0.00 | 0.00 ± 0.00 |
| **10** | 0.00 ± 0.00 | 0.00 ± 0.00 | **1.00** ± 1.10 | **0.33** ± 0.82 | **1.50** ± 0.84 | **0.67** ± 1.03 |
| **11** | 0.00 ± 0.00 | **0.50** ± 1.00 | **0.67** ± 1.03 | 0.00 ± 0.00 | **1.83** ± 1.33 | **1.17** ± 1.33 |
| **12** | 0.00 ± 0.00 | **2.00** ± 1.63 | **2.33** ± 0.82 | **2.00** ± 0.00 | **4.33** ± 1.37 | **3.67** ± 1.03 |
| **13** | 0.00 ± 0.00 | **3.50** ± 1.29* | **3.50** ± 1.38 | **4.17** ± 0.75 | **5.17** ± 1.60 | **5.33** ± 2.50 |
| **14** | 0.00 ± 0.00 | **6.25** ± 2.63* | **4.17** ± 2.32 | **6.00** ± 0.89 | **7.67** ± 1.63 | **6.67** ± 3.83 |
| **15** | 0.00 ± 0.00 | **9.00** ± 1.63* | **6.67** ± 1.75 | **7.83** ± 3.49 | **7.83** ± 0.75 | **7.33** ± 4.80 |
| **16** | 0.00 ± 0.00 | **10.25** ± 2.63* | **10.00** ± 2.68 | **9.83** ± 3.13 | **10.17** ± 3.43 | **9.33** ± 4.18 |
| **17** | 0.00 ± 0.00 | **11.25** ± 4.03* | **11.67** ± 4.03 | **11.17** ± 4.40 | **10.67** ± 3.83 | **10.67** ± 4.84 |
| **18** | 0.00 ± 0.00 | **12.75** ± 3.30* | **10.50** ± 5.54 | **12.67** ± 2.94 | **10.17** ± 4.17 | **10.83** ± 6.62 |
| **19** | 0.00 ± 0.00 | **13.50** ± 3.00* | **10.83** ± 5.71 | **12.33** ± 2.73 | **11.33** ± 2.73 | **11.83** ± 5.27 |
| **20** | 0.00 ± 0.00 | **14.00** ± 4.83* | **11.17** ± 5.78 | **11.17** ± 3.82 | **11.17** ± 3.87 | **11.50** ± 5.96 |
| **21** | 0.00 ± 0.00 | **14.25** ± 2.06* | **10.67** ± 5.92 | **12.67** ± 3.72 | **11.50** ± 3.27 | **10.83** ± 5.42 |
| **22** | 0.00 ± 0.00 | **14.75** ± 1.26* | **11.00** ± 6.00 | **13.33** ± 3.08 | **11.67** ± 3.56 | **12.17** ± 4.88 |
| **23** | 0.00 ± 0.00 | **14.50** ± 1.73* | **11.17** ± 6.34 | **12.83** ± 3.25 | **11.50** ± 4.23 | **11.33** ± 5.28 |
| **24** | 0.00 ± 0.00 | **13.75** ± 2.63* | **11.33** ± 6.31 | **12.67** ± 3.44 | **10.50** ± 3.94 | **11.50** ± 3.89 |
| **25** | 0.00 ± 0.00 | **12.50** ± 2.38* | **10.50** ± 6.02 | **12.33** ± 2.88 | **9.00** ± 4.43 | **11.50** ± 4.97 |

The arthritis score is shown as average ± SD. *(p<0.05) denotes statistically significant differences Control– vs Control+.
